# Patients With Hypoplastic Left Heart Syndrome Have a Shorter Superior Vena Cava

**DOI:** 10.1016/j.atssr.2024.01.016

**Published:** 2024-02-28

**Authors:** Akira Yamaguchi, Shintaro Iwamoto

**Affiliations:** 1 Division of Cardiovascular Surgery, National Center for Child Health and Development, Tokyo, Japan; 2 Biostatistics Unit, Department of Data Science, National Center for Child Health and Development, Tokyo, Japan

## Abstract

**Background:**

The primary treatment for hypoplastic left heart syndrome (HLHS) is the Fontan pathway, which entails performing the Glenn procedure. We hypothesized that the superior vena cava in patients with HLHS was short. As the length of the superior vena cava influences the Glenn procedure, we compared its length between patients with HLHS and those with other congenital heart diseases.

**Methods:**

Patients with HLHS or its variant, patients with ventricular septal defects (VSD), and patients with pulmonary atresia with intact ventricular septum (PA/IVS)—including critical pulmonary stenosis—were enrolled in this study. The effective superior vena cava ratio (ESCVR), which is defined as the inferior border of the left brachiocephalic vein to the superior surface of the right pulmonary artery/height, was measured.

**Results:**

The median ESVCR of the HLHS, VSD, and PA/IVS patients was 12.54 mm/m, 17.96 mm/m, and 18.46 mm/m, respectively. ESVCR of the HLHS group was significantly smaller than that of the other groups (*P* = .0013 vs VSD group, *P* = .0002 vs PA/IVS group).

**Conclusions:**

Patients with HLHS have a relatively short superior vena cava, which may complicate the Glenn procedure.

**In Short**

▪ Patients with hypoplastic left heart syndrome have a shorter superior vena cava than those with other congenital heart conditions.
▪ A short superior vena cava may complicate the Glenn procedure.

The primary treatment for hypoplastic left heart syndrome (HLHS) is the Fontan pathway, which entails performing the Glenn procedure.

The Glenn procedure, which is an interstage surgery during the Fontan pathway, refers to the end-to-side anastomosis between the superior vena cava (SVC) and the pulmonary artery. The Glenn procedure requires a meticulous anastomosis technique to prevent stenosis and thrombus formation.^1^, ^2^, ^3^

We hypothesized that patients with HLHS had a shorter SVC than those with other congenital heart diseases. A short SVC may potentially complicate the Glenn procedure. This study aims to analyze and compare the length of the SVC among 3 groups of patients with different congenital heart diseases.

## Material and Methods

The ethics committee of the National Center for Child Health and Development approved our study on August 5, 2022 (number: 2022-055). The need for parental consent was waived. Data of surgeries performed from March 2009 to January 2022 were retrieved from the database of the National Center for Child Health and Development. We identified patients with HLHS and its variant—categorized into the HLHS group—who underwent the Glenn procedure, patients with ventricular septal defect (VSD) who underwent intracardiac repair, and patients with pulmonary atresia with intact ventricular septum (PA/IVS) or critical pulmonary stenosis—categorized into the PA/IVS group—who underwent the Glenn procedure, palliative right ventricular outflow tract reconstruction, or intracardiac repair. Among these patients, those who had a normal position of the great vessels based on preoperative computed tomography (CT) were eligible for this study. The criteria for CT in patients with VSD were based on physician’s discretion. Patients with specific morphologic features that may influence the ESVCR, such as situs ambiguous, bilateral SVC, and right aortic arch, were excluded.

**Figure 1:**
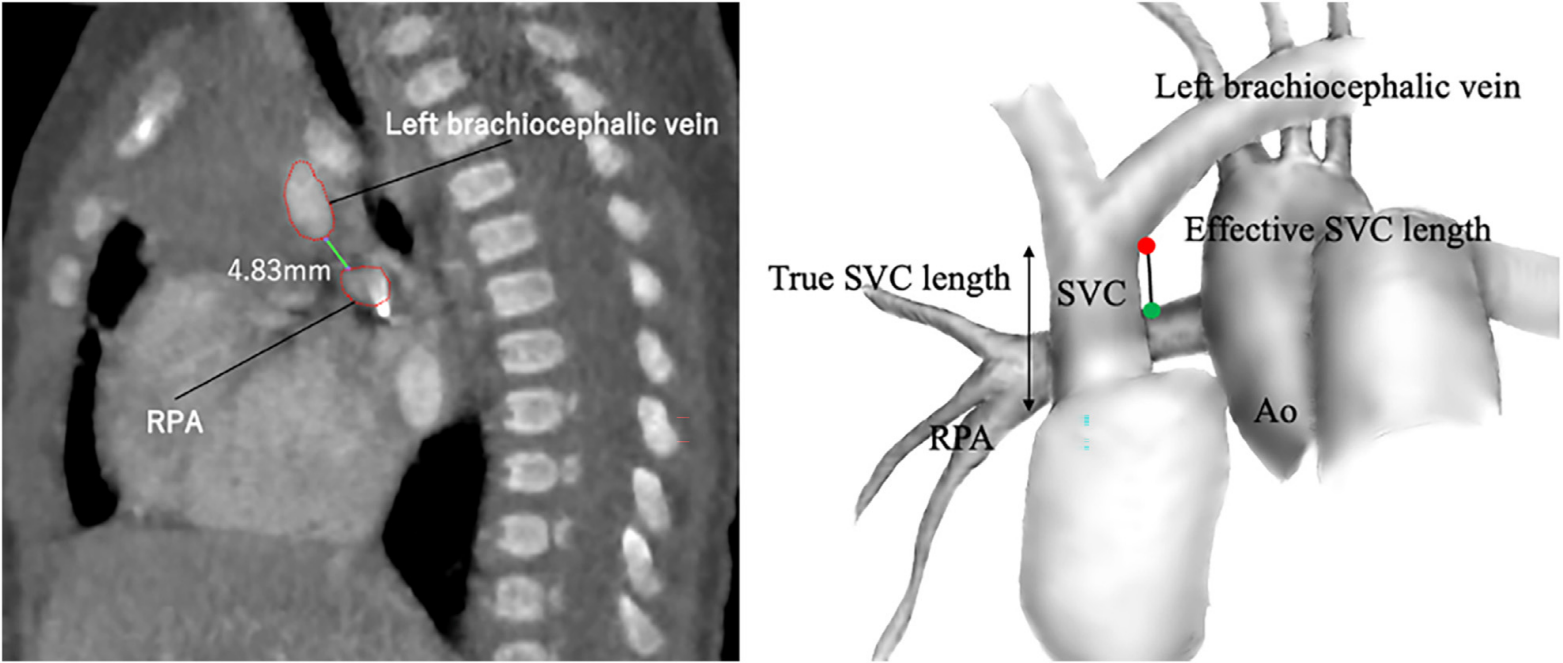
Sagittal computed tomography (CT) image of a patient with hypoplastic left heart syndrome and its variant. Green line in the CT image indicates the effective superior vena cava (SVC) length, which is defined as the distance between the red and green points in the image. (Ao, ascending aorta; RPA, right pulmonary artery.)

We utilized preoperative CT images to measure the length of the SVC. For the SVC length, we introduced the effective SVC length, which represents the length of the SVC that can be used for the Glenn procedure. The effective SVC length was defined as the distance between the inferior border of the left brachiocephalic vein-SVC confluence and the superior surface of the right pulmonary artery, where the Glenn anastomosis would be created. To negate the effects of body size, the effective SVC length in millimeters was divided by the height of the patient in meters; the result was termed the “effective superior vena cava ratio” (ESVCR). The primary outcome of this study was the ESVCR, which was assessed using sagittal CT images (Figure 1). The ESVCRs of the 3 groups were then compared.

We also divided patients with PA/IVS based on treatment pathways: biventricular (biPA/IVS) and single /1.5 ventricular repair (sPA/IVS) groups. Subgroup analysis among HLHS group, biPA/IVS group, and sPA/IVS group was conducted.

For statistical analysis, patients’ demographics were stratified according to diagnosis and presented as median and interquartile range (IQR) for continuous variables and numbers and percentages for categorical variables. Completion of missing values was not performed in this study. The primary endpoint of the study was the ESVCR. As we wanted to determine where the differences were among the 3 groups, we used the Steel-Dwass test for comparison.^4^^,^^5^ The Steel-Dwass test was 2-sided and considered statistically significant when the *P* value was <.05. Box-and-whisker plots were also created for the ESVCR of all groups to visualize the distribution. R software version 4.2.0 (https://www.R-project.org/) was utilized for all statistical analyses.

## Results

Overall, 95 patients (HLHS: n = 29; VSD: n = 40; PA/IVS: n = 26) were identified for the study. Among these, 6, 14, and 2 patients in the HLHS, VSD, and PA/IVS groups, respectively, were excluded. Finally, the HLHS, VSD, and PA/IVS groups included 23, 26, and 24 patients, respectively.

The patient characteristics are shown in Table 1. Five patients with HLHS variant were included in the HLHS group. There were no missing values for the variables shown in Table 1.

**Table 1:** Patient Characteristics of Patients

Characteristic	HLHS	VSD	PA/IVS
n	23	26	24
Male	11 (47.8)	12 (46.2)	12 (50.0)
Age, d	89.00 (76.00-112.50)	122.50 (55.50-234.00)	150.00 (105.50-353.25)
Height, m	0.56 (0.52-0.60)	0.56 (0.51-0.62)	0.63 (0.56-0.74)
Body weight, g	4210.00 (3637.50-4909.00)	4372.50 (3199.00-5987.50)	6530.50 (4593.00-8176.00)
ESVCR, mm/m	12.54 (8.96-17.45)	17.96 (15.92-20.60)	18.46 (16.29-21.68)
AoD, % of normal	37 (28-54)	89 (78-103)	111 (102-119)
Biventricular repair	0 (0.0)	26 (100.0)	6 (25.0)
Genetic disorder	0 (0.0)	6 (23.1)	1 (4.2)

Values are presented as n (%) or median (interquartile range) unless otherwise noted.

AoD, ascending aorta diameter; ESVCR, effective superior vena cava ratio; HLHS, hypoplastic left heart syndrome including its variant; PA/IVS, pulmonary atresia with intact ventricular septum or critical pulmonary stenosis; VSD, ventricular septal defect.

**Figure 2:**
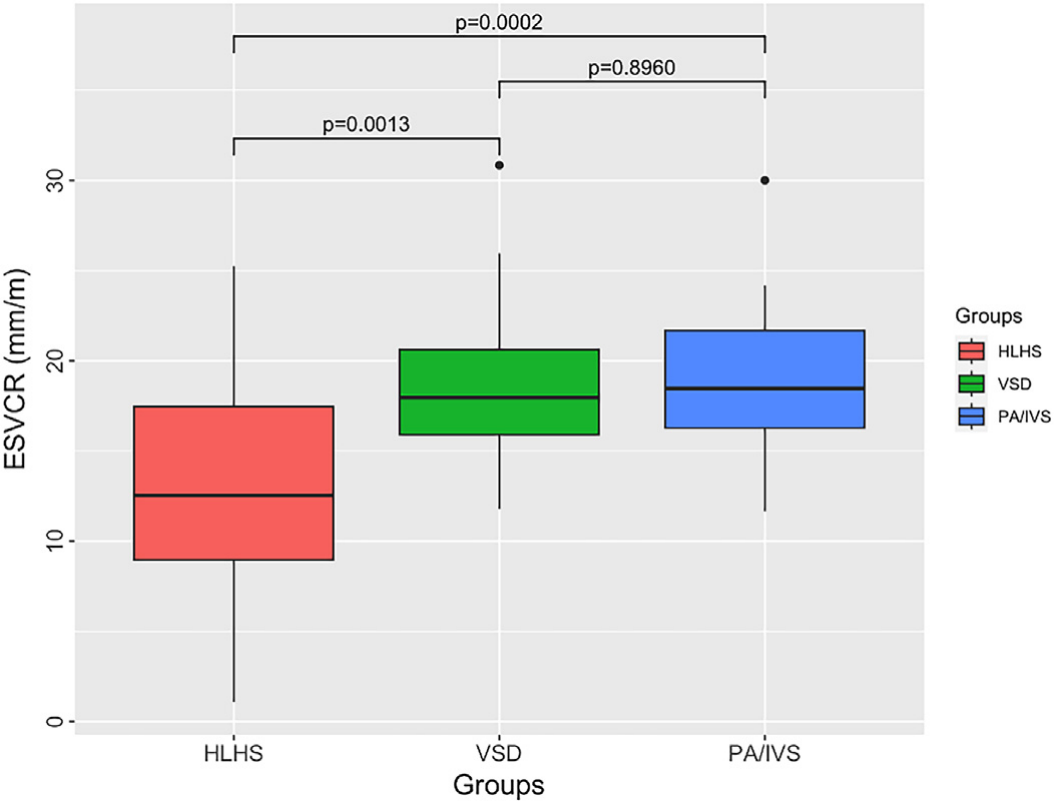
Patients with hypoplastic left heart syndrome and its variant (HLHS) had a significantly smaller effective superior vena cava ratio **(**ESVCR) than patients with ventricular septal defect (VSD) and pulmonary atresia with intact ventricular septum or critical pulmonary stenosis (PA/IVS).

The median and IQR ESVCR is shown in Table 1. The ESVCR of the HLHS group was significantly smaller than that of the VSD and PA/IVS groups (Figure 2). The difference in the ESVCR between the VSD and PA/IVS groups was not significant.

**Figure 3:**
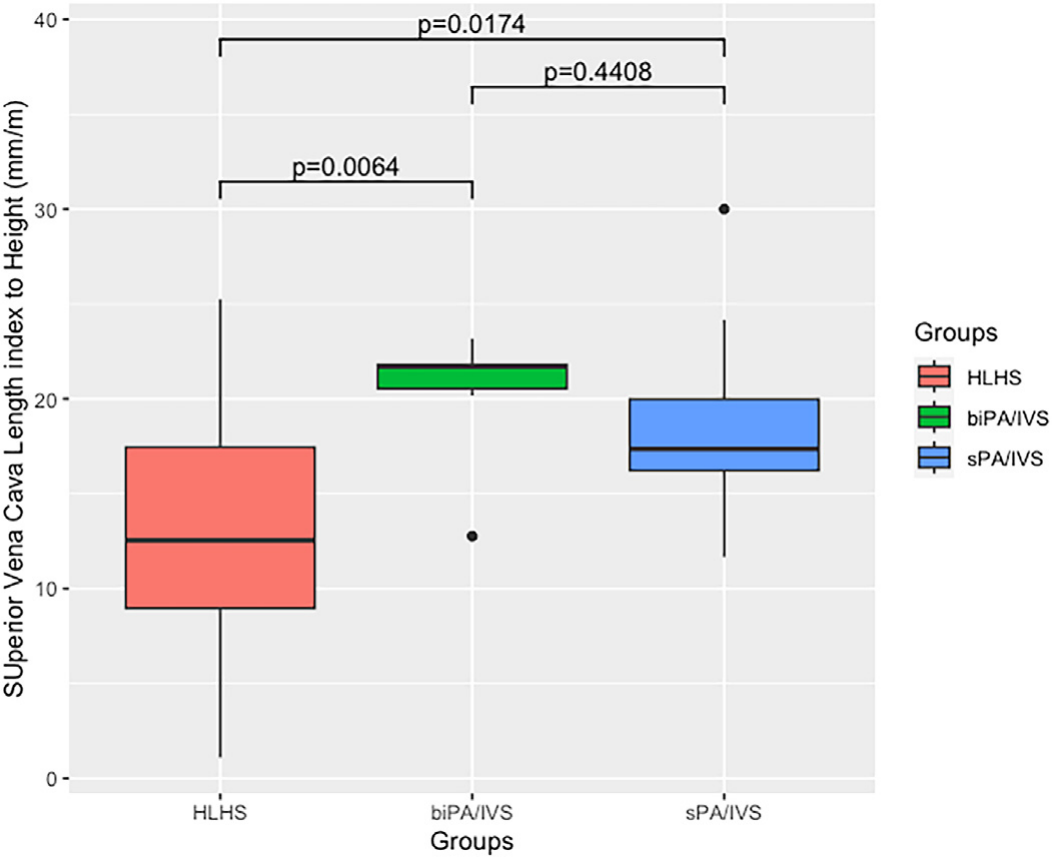
The hypoplastic left heart syndrome and its variant (HLHS) group had a significantly smaller effective superior vena cava ratio **(**ESVCR) than pulmonary atresia with intact ventricular septum or critical pulmonary stenosis on biventricular repair track (biPA/IVS) and pulmonary atresia with intact ventricular septum or critical pulmonary stenosis on single/1.5 ventricular repair track (sPA/IVS) groups.

As noted earlier patients with PA/IVS were divided into 2 subgroups (biPA/IVS: n=6, sPA/IVS: n=18). The median and IQR ESVCR for these subgroups is shown in Supplemental Table 1. All patients in the sPA/IVS group underwent Glenn procedure. The ESVCR of the HLHS group was significantly smaller than that of both the biPA/IVS (median: 21.70 mm/m, IQR: 20.54-21.80mm/m) and sPA/IVS (median: 17.36mm/m, IQR: 16.22-19.98 mm/m) groups (Figure 3). The difference in ESVCR between the biPA/IVS and sPA/IVS groups was not significant.

## Comment

In this study, the ESVCR in patients with HLHS was significantly smaller than those with other congenital heart diseases. This result may be attributed to the left brachiocephalic vein merging into the proximal SVC. Chen and associates^6^ postulated that the development of the great arteries exerts direct pressure on the upper transverse capillary plexus, halting the left brachiocephalic vein from merging into the correct site. Other researchers have also suggested that the left brachiocephalic vein develops when reduced spatial hindrance is present.^7^^,^^8^ This hypothesis may explain the left brachiocephalic vein merging into the proximal SVC due to the spatial allowance provided by the hypoplastic ascending aorta.

For the embryogenic interaction between the left brachiocephalic vein and the ascending aorta, we hypothesized that the length of the SVC was correlated with the ascending aortic diameter (AoD). Based on this hypothesis, we enrolled patients with different AoD as follows: patients with HLHS (small-sized AoD); patients with VSD (normal-sized AoD); and patients with PA/IVS (large-sized AoD). Although the correlation between the ESVCR and AoD was calculated, it was insignificant and inconclusive in this study. A larger sample size is needed to evaluate the correlation between the length of the SVC and AoD.

We also hypothesized that patients on single ventricular repair track could have a shorter SVC regardless of their diagnosis. Therefore, we conducted a subgroup analysis comparing the HLHS group and the sPA/IVS group. ESVCR was significantly smaller in both stratifications by diagnosis and procedure type. These findings led to the conclusion that patients with HLHS intrinsically have a shorter SVC.

### Procedural Modifications for the Glenn Procedure in Patients With HLHS at Our Institution

At our institution, most patients with HLHS undergo bilateral pulmonary artery banding within 1 week of age followed by prostaglandin infusion. The Norwood and Glenn procedure is then performed at approximately 12 weeks of age in most patients.

Herein, we describe the techniques of the Glenn procedure for patients with a short SVC. Procedural modifications include division of the right internal thoracic vein draining into the SVC and venous cannulation cephalad at the confluence of the bilateral brachiocephalic veins with optional temporary occlusion of either brachiocephalic vein.

The right internal thoracic vein drains into the SVC; it is then divided and mobilized. Tearing the internal thoracic vein could form a hematoma around the SVC and may obstruct the surgical field.

We usually perform venous cannulation at a cephalad site, for example, at the confluence of the bilateral brachiocephalic veins. An appropriate cannulation site secures the effective SVC length for the Glenn procedure while allowing for adequate venous drainage. If an adequate SVC length cannot be secured, a more cephalad site for venous cannulation should be considered as well as temporary occlusion of the left brachiocephalic vein.

These techniques may be effective for patients with a short SVC. Additionally, patients with HLHS are potential candidates for heart transplantation even after Fontan repair. These techniques may potentially be applied in heart transplantation procedures for patients with HLHS.

### Limitations

The ESVCR was measured in open-label fashion by a single person using CT images, which may result in inter-observer variance. Additionally, the ESVCR is not an established marker for the maneuverability of the SVC in the Glenn procedure. Moreover, the influence of a short SVC on the technical difficulty of the Glenn procedure has not been established nor evaluated in this study. CT images capture random systolic or diastolic phases, which may influence the measurement of the length or diameter of objects. The follow-up CTs were not available in most patients after completing treatments, and the trajectory of the length of the SVC remains unknown. Finally, CT was not routinely performed for all patients with VSD. Its criteria depend on physician’s discretion, the presence of concomitant lesions in other organs, and era. We attemped to minimize the selection bias by excluding the specific morphology and setting the outcome as a ratio; however, selection bias in VSD patients still remains.

### Conclusion

Patients with HLHS have a shorter SVC than those with other congenital heart conditions. A short SVC may complicate the Glenn procedure.
