# Supplementary material for: Patients With Hypoplastic Left Heart Syndrome Have a Shorter Superior Vena Cava
Source: Ann Thorac Surg Short Rep. 2024 Feb 28;2(3):385–9. doi: 10.1016/j.atssr.2024.01.016 (PMC11708465; doi:10.1016/j.atssr.2024.01.016)
Supplement: Supplemental Table 1 [file mmc1.docx]

**Supplemental Table 1**

|  | HLHS | biPA/IVS | sPA/IVS |
| --- | --- | --- | --- |
| n | 23 | 6 | 18 |
| Male (%) | 11 (47.8) | 3 (50.0) | 9 (50.0) |
| Age (days)  (Median [IQR]) | 89.00  [76.00, 112.50] | 148.50  [104.75, 371.50] | 150.00  [106.00, 308.25] |
| ESVCR (mm/m)  (Median [IQR]) | 12.54  [8.96, 17.45] | 21.70  [20.54, 21.80] | 17.36  [16.22, 19.98] |
| AoD (% of Normal)  (Median [IQR]) | 37.00  [27.50, 53.50] | 119.00  [115.25, 124.25] | 107.50  [96.75, 115.00] |
| Genetic disorder (%) | 0 (0.0) | 1 (16.7) | 0 (0.0) |

AoD: ascending aorta diameter, ESVCR: effective superior vena cava ratio, HLHS: hypoplastic left heart syndrome including its variant, IQR: interquartile range, biPA/IVS: patients with pulmonary atresia with intact ventricular septum or critical pulmonary stenosis who underwent biventricular repair, sPA/IVS: patients with pulmonary atresia with intact ventricular septum or critical pulmonary stenosis who underwent single/1.5 ventricular repair
